# Source Control and Antibiotics in Intra-Abdominal Infections

**DOI:** 10.3390/antibiotics13080776

**Published:** 2024-08-16

**Authors:** Raffaele Bova, Giulia Griggio, Carlo Vallicelli, Giorgia Santandrea, Federico Coccolini, Luca Ansaloni, Massimo Sartelli, Vanni Agnoletti, Francesca Bravi, Fausto Catena

**Affiliations:** 1 General, Emergency and Trauma Surgery Department, Bufalini Hospital, 47521 Cesena, Italy; giulia.griggio@outlook.it (G.G.); sgiorsg@gmail.com (G.S.); fausto.catena@auslromagna.it (F.C.); 2 General, Emergency and Trauma Surgery Department, Pisa University Hospital, 56124 Pisa, Italy; federico.coccolini@gmail.com; 3 Department of General and Emergency Surgery, Policlinico San Matteo, 27100 Pavia, Italy; aiace63@gmail.com; 4 Department of Surgery, Macerata Hospital, 62100 Macerata, Italy; massimosartelli@gmail.com; 5 Anesthesia, Intensive Care and Trauma Department, Bufalini Hospital, 47521 Cesena, Italy; vanni.agnoletti@auslromagna.it; 6 Healthcare Administration, Santa Maria delle Croci Hospital, 48121 Ravenna, Italy; francesca.bravi@auslromagna.it

**Keywords:** source control, antibiotic, intra-abdominal infections, peritonitis, antimicrobial, sepsis

## Abstract

Intra-abdominal infections (IAIs) account for a major cause of morbidity and mortality, representing the second most common sepsis-related death with a hospital mortality of 23–38%. Prompt identification of sepsis source, appropriate resuscitation, and early treatment with the shortest delay possible are the cornerstones of management of IAIs and are associated with a more favorable clinical outcome. The aim of source control is to reduce microbial load by removing the infection source and it is achievable by using a wide range of procedures, such as definitive surgical removal of anatomic infectious foci, percutaneous drainage and toilette of infected collections, decompression, and debridement of infected and necrotic tissue or device removal, providing for the restoration of anatomy and function. Damage control surgery may be an option in selected septic patients. Intra-abdominal infections can be classified as uncomplicated or complicated causing localized or diffuse peritonitis. Early clinical evaluation is mandatory in order to optimize diagnostic testing and establish a therapeutic plan. Prognostic scores could serve as helpful tools in medical settings for evaluating both the seriousness and future outlook of a condition. The patient’s conditions and the potential progression of the disease determine when to initiate source control. Patients can be classified into three groups based on disease severity, the origin of infection, and the patient’s overall physical health, as well as any existing comorbidities. In recent decades, antibiotic resistance has become a global health threat caused by inappropriate antibiotic regimens, inadequate control measures, and infection prevention. The sepsis prevention and infection control protocols combined with optimizing antibiotic administration are crucial to improve outcome and should be encouraged in surgical departments. Antibiotic and antifungal regimens in patients with IAIs should be based on the resistance epidemiology, clinical conditions, and risk for multidrug resistance (MDR) and Candida spp. infections. Several challenges still exist regarding the effectiveness, timing, and patient stratification, as well as the procedures for source control. Antibiotic choice, optimal dosing, and duration of therapy are essential to achieve the best treatment. Promoting standard of care in the management of IAIs improves clinical outcomes worldwide. Further trials and stronger evidence are required to achieve optimal management with the least morbidity in the clinical care of critically ill patients with intra-abdominal sepsis.

## 1. Background

Intra-abdominal infections (IAIs) account for a major cause of morbidity and mortality and healthcare expenditure worldwide. They constitute the second leading cause of septic cases in critically ill patients, affecting approximately 5% of patients admitted to an intensive care unit (ICU), and the second most common sepsis-related death after lower respiratory infections, reporting a hospital mortality of 23–38% [1,2,3].

Intra-abdominal infections include a wide range of pathological conditions, from simple cases of appendicitis to fecal peritonitis, and involve lesions of all intra-abdominal organs [4,5]. The severity of the disease can vary from mild cases to severe intra-abdominal sepsis (IAS), defined as organ dysfunction with SOFA ≥ 2 (Table 1) due to intra-abdominal infection. According to published research, people who satisfy these criteria had lengthy hospital stays and increased risk of death [6,7].

Clinical manifestations are widely varied and IAS, especially if poorly managed, can cause immune response deregulation leading to septic shock. Leligdowicz et al. [8] showed that septic shock was the highest hospital mortality due to intra-abdominal infections, second only to ischemic bowel disease, while the lowest was associated with obstructive uropathy-associated urinary tract infection.

Adequate workup strategies to deal with intra-abdominal infections include a prompt identification of the sepsis source, appropriate resuscitation, and early treatment with the shortest delay possible. Those strategies represent the cornerstones of management of IAIs and are associated with a more favorable clinical outcome.

A prompt diagnosis of severe IAS often requires an adequate clinical evaluation, laboratory tests, and imaging exams, such as abdominal ultrasound or Computed Tomography (CT), which are among the most used diagnostic procedures in emergency settings [9]. A multidisciplinary approach is required for the management of abdominal sepsis that is often needed for an early and effective surgical source control and an adequate antibiotic therapy. Stratifying patients in risk/benefit categories regarding interventions with potential serious effects and iatrogenic morbidity should be crucial in best managing intra-abdominal sepsis [2].

Source control of intra-abdominal infections is achievable by using a wide range of technical procedures. The aim is to reduce the microbial load by removing infection sources. It can be feasible by definitive surgical removal of anatomic infectious foci, percutaneous drainage and toilette of infected collections, decompression, and debridement of infected and necrotic tissue or device removal, providing for the restoration of anatomy and function [2,10,11,12].

As suggested by Surviving sepsis campaign: international guidelines for management of sepsis and septic shock 2021 [13], sepsis and septic shock are medical emergencies which require an immediate treatment and resuscitation. Therefore, the early recognition of the septic source is of primary concern because ongoing severe IAS requires prompt administration of empiric broad-spectrum antibiotic therapy. For adults, this should be administered within 3 h of first recognition of possible sepsis without shock, or immediately/within 1 h of the time when possible septic shock or a high likelihood for sepsis is first recognized. At the same time, adequate fluid and/or vasopressor agents for resuscitation should be administered with the aim of an initial target mean arterial pressure (MAP) of 65 mm Hg.

A multidisciplinary approach combining source control, antibiotic therapy, and resuscitation is necessary to provide the most effective treatment of complicated intra-abdominal infections. In 2023, global clinical guidelines regarding source control in emergency general surgery were proposed [2], in which the authors showed their opinions about the concepts and operational adequacy of source control in intra-abdominal infections, with the aim to improve patient clinical care and encourage future improvements.

## 2. Intra-Abdominal Infections

An unregulated host response to infection results in sepsis, which is defined by the Sepsis-3 criteria as a life-threatening organ malfunction with an increase of at least two points in the Sequential Organ Failure Assessment (SOFA) score. Rather, the term “septic shock” refers to a prolonged hypotension brought on by sepsis that needs to be maintained with vasopressors in order to maintain a mean arterial pressure (MAP) of more than 65 mmHg or a lactate level greater than 2 mmol/L even after appropriate volume resuscitation [14]. Individuals should be deemed as at risk of sepsis if they exhibit two or more items encompassed in the Quick SOFA (qSOFA):

(1) Tachypnea (>22 respiratory acts per minute);
(2) Hypotension (systolic blood pressure < 100 mmHg);
(3) Alteration of the state of consciousness (Glasgow Coma Scale < 15) [15].

IAIs are a significant contributor to morbidity and death. Prompt diagnosis, appropriate resuscitation, early antimicrobial (AM) therapy initiation, early and effective source control, and frequent evaluations of the patient’s clinical response to modify the management plan are the key components of an effective treatment of IAIs [16]. A number of factors need to be taken into account when assessing intra-abdominal infections, including the patient’s clinical state, the suspected pathogens, the anatomical extent, and local patterns of antibiotic resistance. Intra-abdominal infections can be categorized as uncomplicated if they affect just one organ and do not spread to the peritoneum, or complicated if the infection spreads to the peritoneum, causing localized or diffuse peritonitis [16]. Peritonitis can be both sterile and infectious, depending on the underlying disease. Infectious peritonitis is described as follows [4]:

- Primary: diffuse bacterial infection without loss of integrity of the gastrointestinal tract (typical of cirrotic ascetic patients or patients who undergo peritoneal dialysis); it usually requires no surgical treatment.
- Secondary (the most common ones): derived from loss of integrity of the gastrointestinal tract.
- Tertiary: recurrent peritoneal infection which occurs more than 48 h after apparently successful and adequate surgical source control of secondary peritonitis (usually associated with multidrug-resistant organisms, common in immunocompromised patients, associated with high morbidity and mortality).

Furthermore, infections in emergency surgery can be classified into community-acquired (CA-IAIs) and healthcare-associated infections (HA-IAIs), which are infections that occur in a patient during the course of care in a hospital or other healthcare facilities but were not present or incubating at the time of admission. This distinction is useful in defining presumed resistance patterns and identifying patients at higher risk of infections caused by multidrug-resistant microorganisms (surgical site infections, catheter-associated urinary infections, hospital-acquired pneumonia, ventilator-associated pneumonia, central venous catheter-associated bloodstream infections, and Clostridiodes difficile infections) [17,18]. Infections related to medical care have been linked to lengthier hospital stays for patients, requiring second-line or broader spectrum and more expansive antimicrobials, and put more strain on the healthcare system.

Early clinical evaluation is mandatory in order to optimize diagnostic testing and establish a therapeutic plan. Scores may be useful in clinical practice to assess the severity and the prognosis of the disease, helping in the selection of treatment options and patient management. Scoring systems can be divided in two groups:

- General organ failure severity scores: they assess various organ systems for the presence of dysfunction and are used in sepsis and other causes of multi-organ failure (e.g., APACHE II score, SAPS score, and SOFA score) [19,20,21].
- Peritonitis-specific (surgical) scores: calculated before and during surgery; they often include characteristics of the peritoneal contamination (for instance: P-Possum, MPI score, PIA score, and WSES complicated IAIs score from the WISS study) [22,23,24,25].

## 3. Principles of Source Control

Clinical assessment, medical examinations, and imaging (abdominal ultrasound or CT scan) need to be carried out quickly to diagnose IAIs. It is crucial to promptly manage the source of the infection. The major goals of intervention of IAIs are to determine the cause, control the origin of the intra-abdominal sepsis through drainage of abscesses or infected fluid collections, debridement of necrotic or diseased tissues, and definitive control of the contamination source [18]. Source control should be undertaken as soon as possible in patients with extensive peritonitis and no later than 24 h in individuals with a localized illness if appropriate antimicrobial therapy is administered [11].

Blood culture sampling should be performed prior to beginning antibiotic medication.

In hemodynamically unstable patients, source control can be postponed, but operative therapy is still the preferred treatment for intra-abdominal infections. It includes percutaneous drain or surgical treatment: well-localized fluid collections of adequate density can be drained percutaneously [26,27], while surgical source control includes resection or suture of diseased viscus, removal of the infected organ, debridement of necrotic tissue, resection of ischemic bowel, and repair/resection of traumatic lesions.

Laparoscopic peritoneal washing in complex acute diverticulitis is debatable, and its efficacy has not been established [28]: in most circumstances, in patients with complicated acute diverticulitis, percutaneous abscess drainage or surgical resection are preferable. Selected individuals with severe diverticulitis (abscesses less than 4 cm), a peri-appendicular tumor, or a perforated peptic ulcer who respond to AM treatment and other supportive measures can be treated without source control. Abscesses can be treated with intravenous antibiotics alone or with a percutaneous drain, depending on their size (a maximum diameter of 3–6 cm is typically allowed for antibiotic treatment) [29,30]. Antibiotics alone may be administered in individuals with early, non-perforated appendicitis [31], although in some patients with complicated appendicitis, initial non-operative treatment may be considered [32,33].

In certain septic patients, damage control surgery may be an option to allow early draining of any residual infection and management of any persisting source of infection, delaying definitive intervention until the patient is hemodynamically stable [34,35]. Negative pressure can be used to reduce the time to definitive abdominal closure, minimizing issues related to open abdomen (skin trauma, abdominal wall retraction, loss of domain, or bowel edema). Conversely, constant negative pressure leads to an increase in the occurrence of enteric fistulae, at approximately 14% [36,37,38].

### Timings of Source Control and Patients Stratification

The general idea of “as soon as possible” in source control is supported by numerous studies, the correct time frames range from 7 to 24 h in patients with IAIs without systemic inflammations [39,40,41].

The current guidelines published in the World Journal of Emergency Surgery propose three levels of SC urgency [2]:

- Emergent source control (high mortality risk, severe physiological derangement caused by the acute disease): SC is mandatory and must be initiated as soon as feasible.
- Urgent source control: the intervention can be delayed up to 24 h to improve the clinical status of the patient (fluid resuscitation and broad-spectrum antibiotic therapy).
- Delayed source control (stable patient, low risk): the SC can be delayed until the infectious process is well defined to decrease the chance of collateral operation injury.

Furthermore, a stratification of the risk for patient comorbidity should always be taken into consideration while developing a source control (SC) strategy (Table 2).

In patients with compromised immunological conditions, source control may become more complex, necessitating the involvement of multidisciplinary teams to achieve optimum results. A multitude of further conditions, surgical risk factors, and physiological states increase the likelihood of complications from intra-abdominal infections and call for source control adaptation, such as hypoalbuminemia, advanced age, high body mass index (BMI), smoking, diabetes mellitus, and ischemia secondary to vascular disease [42].

## 4. Antibiotic Management

Severe intra-abdominal infections need multidisciplinary management in which the appropriate administration of an AM regimen is a paramount role. Sepsis prevention and infection control protocols combined with optimizing antibiotic administration are crucial to improve outcome and should be encouraged in surgical departments; current evidence suggests emergency surgery settings should aim to provide a sepsis team with figures, clinical experience, and adequate training to improve the management of IAIs [43]. In 2017, the Global Alliance for Infections in Surgery Working Group released a position article in which they published a global declaration on the appropriate use of AM agents across the surgical pathway [44].

Antibiotics and antifungal regimens in patients with IAS should be based on the resistance epidemiology, clinical conditions, and risk for multidrug resistance (MDR) and Candida spp. infections [45,46,47]. Regardless of the anatomical source of sepsis, early and adequate empirical broad-spectrum AM treatment can have a significant effect on the clinical outcome, especially in critically ill patients, and it should be started as soon as possible in cases of septic shock. The Surviving Sepsis Campaign International Guidelines 2021 [13] recommended administering antimicrobial therapy immediately and ideally within 1 h of recognition for adults with possible septic shock or very probable sepsis. In the case of possible sepsis but without shock, a prompt assessment of the likelihood of infections versus non-infectious causes of acute illness has been recommended, and this should be achieved within 3 h of presentation so that timely AM therapy can be provided if the likelihood of sepsis is considered to be significant. Alternatively, for adult patients with an unlikely infection and without shock, deferring antimicrobial therapy while continuing to closely monitor the patients is suggested.

The adequacy and effectiveness of the therapy should be reassessed daily, while also paying particular attention to the appearance of possible side effects, to avoid toxicity, minimize cost, and reduce the growth of resistant bacterial and fungal strains. In surgical settings, the physicians can achieve this by clinical monitoring and the use of laboratory tests, including white blood cells count, C reactive protein, and kidney and liver function tests. The use of highly sensitive biomarkers of surgical infection such as procalcitonin is reasonable, and it can be a key tool to guide the duration and cessation of antibiotics [48]. Empiric broad-spectrum AM therapy should be de-escalated or changed once antibiograms are available and/or clinical improvement is achieved. The prolonged use of antibiotics appears to be one of the main causes of the rise of antimicrobial resistance worldwide. Single doses have been shown to have the same effect on clinical outcome as multiple doses in the management of uncomplicated IAS, such as uncomplicated appendicitis or acute cholecystitis; in particular, post-operative antibiotic therapy is not required if source control is adequate. In the setting of complicated IAS and in patients with post-operative intra-abdominal infections, a short course of antibiotic therapy is an effective and safe option [45,47,49,50,51]. Montravers et al. [52] showed that short-course antibiotic treatment in critically ill ICU patients with post-operative intra-abdominal infections reduces antibiotic exposure without differences in terms of 45-day mortality (rate difference 0.038, 95% CI—0.013 to 0.061). In their multicenter prospective randomized trial, they evaluated the efficacy and safety of 8-day versus 15-day antibiotic therapy (15 [6,7,8,9,10,11,12,13,14,15,16,17,18,19,20] vs. 12 [6,7,8,9,10,11,12,13] days; *p* < 0.0001), further proving that treatments do not differ in terms of hospital and ICU length of stay, reoperation, and appearance of MDR infections rate, so the continuation of antibiotics until day 15 is not associated with clinical benefits.

Therapy continuation in patients with ongoing signs of infection should be tailored to each individual, with decisions on whether to withhold, change, or stop antimicrobial therapy being based on clinical judgment and laboratory tests. In settings with patients with prolonged signs of infection after source control, it is reasonable to consider performing further diagnostic investigations to exclude the presence of other uncontrolled and/or unknown infectious foci which require source control treatment.

Antibiotic agents are too often inappropriately employed, without consideration of local resistance epidemiology and host factors as individual risk factors for multi-resistant organisms and the clinical status of patients. Before the administration of antibiotics, especially in critically ill patients, cultures should be performed. For patients with community-acquired IAIs at risk for MDR, or hospital-acquired IAIs, and for critically ill patients, performing checks for microbiological specimens from the anatomical site of infection is always recommended because it can allow to expand or de-escalate the AM regimen based on the intraperitoneal specimens. The choice of antibiotic regimen must equally consider pharmacokinetic (PK) and pharmacodynamic (PD) principles. Prescribing an adequate dose according to the most appropriate route and mode of administration of an antimicrobial regimen optimizes the likelihood of achieving the desired PK/PD targets and maximizes the exposure and effectiveness of AM agents [53]. Knowledge of AM drugs’ properties, including rate of bactericidal action, inhibition of growth, and time-dependent versus concentration-dependent activity, is needed for achieving better clinical outcomes. Antibiotics need to reach an anatomical site of action outside of blood circulation, and suboptimal target site concentrations can lead to therapeutic failure, especially in the case of bacteria with a higher in vitro minimal inhibitory concentration (MIC). The differential tissue distribution should always be evaluated when prescribing antibiotics because drug-related factors and host disease state can contribute to altering it. Patients with impaired renal function need lower than standard doses of renally excreted agents, while higher than standard doses are needed to achieve optimal concentrations in patients with glomerular hyperfiltration [54]. In IAS, the pathophysiology of sepsis should be considered because it can lead to the “third spacing phenomenon”, or a dilution effect that is particularly important for hydrophilic antibiotics (e.g., beta-lactams, aminoglycosides, and glycopeptides) for which higher than standard loading doses are required to achieve optimal exposure at the sepsis source. However, for lipophilic drugs (e.g., fluoroquinolones and tetracyclines) standard dosages are usually sufficient to achieve adequate loading, even in patients with sepsis or septic shock. [45,54]. For drugs characterized by concentration-dependent activity, such as aminoglycosides, the peak plasma concentration is closely associated with AM effectiveness, so they should be administered once daily or with the least possible number of daily administrations to obtain high peak serum concentration. Alternatively, beta-lactams present time-dependent activity, so their effectiveness is associated with the amount of time the drug concentration is maintained above the MIC.

In recent decades, antibiotic resistance has become a global health threat caused by inappropriate antibiotic regimens, inadequate control measures, and infection prevention. Resistant Gram-negative infections are becoming prevalent, but the spread of fungal infections has also increased more recently because of the rising size of the at-risk population, which includes immunocompromised patients, such as patients with human immunodeficiency virus (HIV) infection, patients with cancer, baseline pulmonary or hepatic disease, organ transplant, corticosteroid use, critically ill patients infected in the ICU, patients hospitalized for more than 7 days, or those who have received recent antimicrobial therapy. The Infectious Diseases Society of America coined the term “ESKAPE pathogens” to refer to a group of nosocomial pathogens, encompassing both Gram-positive and Gram-negative species, including *Enterococcus faecium*, *Staphylococcus aureus*, *Klebsiella pneumoniae*, *Acinetobacter baumannii*, *Pseudomonas aeruginosa*, and *Enterobacteriaceae* resistant to carbapenem (CRE) (Table 3), which are currently common causes of nosocomial infections because of their characteristic ability to “escape” the effect of antibiotic agents as multidrug- (MDR), extensive-drug- (XDR), and pan-drug-resistant (PDR) bacteria [55,56].

Among resistant nosocomial microorganisms, several studies showed a significant morbidity in post-operative IAIs associated with enterococcal infections, and that the appearance of strains characterized by glycopeptide resistance became worse with the control of these pathogens [57,58]. Carbapenem-resistant Enterobacterales (CRE), such as Klebsiella Pneumoniae and Escherichia Coli, are another wide order of different pathogens that developed resistance to the group of AMs called carbapenems and are increasingly among the most common cause of infections in healthcare settings, associating with significant mortality especially in immunocompromised patients [59]. Among Enterobacterales, some strains developed a resistance mechanism, becoming capable of producing enzymes that break down and hydrolyze a wide range of beta-lactams including penicillins, 3th cephalosporins, and aztreonam. They are known as extended-spectrum beta-lactamases (ESBLs) bacteria, and this resistance made ESBL infections rapidly prevalent in nosocomial and also in community-acquired infections. Few antibiotic options are available for the treatment (e.g., intravenous carbapenems) because of their possible resistance to multiple antibiotic agents, including fluoroquinolones and aminoglycosides. The main risk factors for ESBL include previous colonization by ESBL within the last 90 days, further broad-spectrum antibiotics for at least 5 days within the last 90 days, or hospitalization for 48 h within the last 90 days [45,60].

IAIs present different clinical spectra globally and have a wide geographical variation. Empiric broad-spectrum antibiotic stewardship for intra-abdominal infections require often drugs characterized by activity especially against aerobic streptococci, Gram-negative bacteria and obligate anaerobic pathogens; in at risk-conditions, additional AM agents could be needed to cover resistant or opportunistic microorganisms such as *Candida* spp. [10]. AM therapy may require single or multiple antibiotic regimens depending on several pathogens and host factors.

Combinations of beta-lactam/beta-lactamase inhibitors such as amoxicillin/clavulanate, ticarcillin/ clavulanate, or piperacillin/tazobactam have been widely employed in the treatment of IAIs because of their activity against Gram-negative, Gram-positive, and anaerobic microorganisms. The growing rates of antimicrobial resistance among *E. Coli* and other Enterobacteriaceae to amoxicillin/clavulanate and other AM drugs used against aerobic Gram-negative bacteria, such as fluoroquinolones (moxifloxacin, ciprofloxacin, levofloxacin), limited the use of these drugs for IAIs therapy, while piperacillin/tazobactam or third generation-cephalosporins comminated with metronidazole could represent a good chance for empiric broad-spectrum treatment of non-severe IAIs [45,46,47]. For the management of complicated intra-abdominal infections, an amazing new option is represented by ceftazidime/avibactam and ceftolozane/tazobactam in combination with metronidazole. Ceftazidime/avibactam is employed especially to treat MDR Gram-negative pathogens with the aim to preserve carbapenems, whereas ceftolozane/tazobactam have good activity against Klebsiella pneumoniae Carbapenemase (KPC) and OXA 48-like b-lactamases, including OXA-48 itself, enterobacteriales [45,61]. The use of carbapenems is the first choice to treat ESBL infections and it should be limited because of the rising carbapenems resistance [47]. Imipinem/cilastatin, meropenem and doripenem provide good activity against particular Gram-negative non-fermenting species, including Pseudomonas aeruginosa and Acinetobacter baumannii [45]. The higher rate of toxic side effects in other antibiotics, including nephrotoxicity and the ototoxicity of aminoglycosides, tigecycline, and eravacycline, limited their employment for the routine empiric management of IAIs, but they are a viable option in the setting of complicated IAIs especially in the case of beta-lactams allergies or in combination with the latter in the setting of critically ill patients with suspected MDR infections [45,47]. Empirical antifungal treatment, such as that against Candida spp., is not recommended for community-acquired IAIs, excluding immunocompromised patients. Antifungal resistance is a problem that is spreading globally, particularly for Candida glabrata, Aspergillus fumigatus and the new emerging MDR species Candida auris [44]. According to the Clinical practice guideline for the management of candidiasis: 2016 update by the Infectious Diseases Society of America, empiric antifungal therapy should be reserved for patients with evident intra-abdominal signs of infection or in cases where there is a significant risk factor of developing Candida peritonitis, including recent abdominal surgery, tertiary peritonitis due to anastomotic leaks, and necrotizing peritonitis [62].

By using evidence-based clinical pathways for the most common IAIs and standardizing the antimicrobial treatment for particular intra-abdominal infections, the 2021 WSES/GAIS/SIS-E/WSIS/AAST global clinical pathways for patients with intra-abdominal infections are intended to advance global standards of care and facilitate clinical management of IAIs globally [45].

## 5. Conclusions

IAIs are still an important cause of morbidity and mortality and healthcare expenditure worldwide. Their clinical manifestations are various, and if poorly managed, they can cause an immune response deregulation due to interaction between pathogenic microorganisms and the host immune system, leading to sepsis and septic shock.

The management of abdominal sepsis requires a multidisciplinary approach. Early diagnosis, effective source control, appropriate empiric antimicrobial therapy, and prompt resuscitation support are the cornerstones for successful management of IAIs. Several challenges still exist regarding the effectiveness, timing, and patient stratification procedures for source control. Correct empirical broad-spectrum antimicrobial therapy in compliance with guidelines and judicious antibiotic administration are paramount to reduce multidrug resistance and improve clinical outcomes. Antibiotic choice, optimal dosing, and duration of therapy are essential to achieve the best treatment.

Promoting standard of care in IAS management is crucial to improve clinical outcomes worldwide. Further trials and stronger evidence are required to achieve optimal management with the least morbidity in the clinical care of critically ill patients with IAS.

## Figures and Tables

**Table 1:** Sepsis-related organ failure assessment score to describe organ dysfunction/failure.

SOFA Score	1	2	3	4
Respiration				
PaO_2_/FiO_2_, mmHg	<400	<300	<200	<100
			with respiratory support	
Coagulation				
Platelets × 10^3^/mm^3^	<150	<100	<50	<20
Liver				
Bilirubin, mg/dL	1.2–1.9	2.0–5.9	6.0–11.9	>12.0
(μmol/L)	(20–32)	(33–101)	(102–204)	(<204)
Cardiovascular				
Hypotension	MAP < 70 mmHg	Dopamine < 5 or or dobutamine (any dose) ^a^	Dopamine > 5 or nor-/epinephrine ≤0.1	Dopamine > 15 or nor-/epinephrine >0.1
Central nervous system				
Glasgow Coma Score	13–14	10–12	6–9	<6
Renal				
Creatinine, mg/dL	1.2–1.9	2.0–3.4	3.5–4.9	>5.0
(μmol/L) or urine			(300–440)	(>440)
output	(110–170)	(171–299)	or <500 mL/day	or <200 mL/day

^a^ Catecholameine doses in µg/kg/min, admistered for at least 1 h.

**Table 2:** Stratification of the risk for patient comorbidity.

Class A	Healthy patients with no or well-controlled comorbidities and no immunocompromise, where the infection is the main problem
Class B	Patient with major comorbidities and/or moderate immunocompromise but currently clinically stable, in whom the infection can rapidly worsen the prognosis
Class C	Patients with important comorbidities in advanced stages and/or severe immunocompromise, in which the infection worsens an already severe clinical condition

**Table 3:** “ESKAPE” pathogens.

**E**	Enterococcus Faecium
**S**	Staphylococcus Aureus
**K**	Klebsiella Pneumoniae
**A**	Acinetobacter Baumannii
**P**	Pseudomonas Aeruginosa Cellulitis, Endocarditis, Prostatitis, UTI, Wound infection and concurrent bacteremia Pneumonia, Skin infections, Endocarditis, Ospeomyelitis, Arthritis Bacteremia, Pneumonia, Gastrointestinal tract infections, Osteomyelitis, Endocarditis Ventilator-associated Pneumonia, Bloodstream infection, Wound infections, Meningitis Endocarditis, Pneumonia, UTI, Wound and Skin infections, Osteomyelitis
**E**	Enterobacteriaceae resistant to carbapenem (CRE)

## Data Availability

No new data were created or analyzed in this study.
